# A high-quality draft genome sequence of *Neonectria faginata*, causative agent of beech bark disease of *Fagus grandifolia*

**DOI:** 10.1128/mra.01048-23

**Published:** 2024-01-24

**Authors:** Eric W. Morrison, Tuan A. Duong, Jeff R. Garnas

**Affiliations:** 1 Department of Natural Resources and the Environment, University of New Hampshire, Durham, New Hampshire, USA; 2 Department of Biochemistry, Genetics and Microbiology, Forestry and Agricultural Biotechnology Institute, University of Pretoria, Pretoria, South Africa; Rochester Institute of Technology, United States

**Keywords:** fungi, neonectria, pathogenic fungi

## Abstract

The draft genome of *Neonectria faginata* was sequenced with Oxford Nanopore and Illumina 250 bp paired-end sequencing technologies. The assembled genome was 42.9 Mb distributed over 24 contigs, with N50 of 4.4 Mb and 98.6% BUSCO completeness. This genome sequence will aid in understanding *N. faginata* population structure and ecology.

## ANNOUNCEMENT

Beech bark disease (BBD) of American beech trees (*Fagus grandifolia*) in North America has significant ecological, economic, and aesthetic impacts (1). BBD is referred to as a disease complex as it arises from the interaction among an introduced insect (*Cryptococcus fagisuga*) and at least one of two fungi*, Neonectria faginata* and *N. ditissima*. The dominant causal agent, *N. faginata*, has never been observed outside of the BBD complex (2) raising questions about its origin and potential alternative ecological modes or hosts. We report a high-quality draft genome of *N. faginata* that will aid in advancing understanding of the natural history of this organism through population genetic and comparative genomic approaches.

The sample was collected from the bark of an American beech tree in Penobscot County, Maine, USA (44.83071 N, 68.59962 W) on 31 May 2018. A culture was isolated following the single-ascospore isolation protocol of Stauder et al. (3) on malt-yeast agar medium [1% (wt/vol) malt extract, 0.2% yeast extract, 2% agar, 50 mg L^−1^ streptomycin, and 10 mg L^−1^ tetracycline). DNA was isolated following van Diepen et al. (4), including a 30-min RNase A digestion (ThemoScientific EN0531). Taxonomic identification was performed by sequencing the *TEF1-*α and *RPB2* genes, performing a multiple sequence alignment with reference sequences from Castlebury et al. (2), and confirming that our isolate grouped within a monophyletic *N. faginata* clade in a neighbor joining tree.

Long read sequencing was carried out with Oxford Nanopore Technology (ONT) using the SQK-LSK109 ligation sequencing kit and a MinION Mk1B sequencing device with a MIN106 flow cell. DNA was size selected using a 1:1 mixture of AxyPrep MAG beads (Axygen MAG-PCR-CL-5). Total read count was 959,919 comprising 3.25 Gbp (475,527 reads ≥ 1 kbp totaling 2.97 Gbp). Read N50 was 11,647 bp. Base calling was performed in Albacore 2.3.4 (5).

An Illumina sequencing library was prepared using a Kapa HyperPlus DNA library kit, and 250 bp paired-end sequencing was performed on a HiSeq 2500 with Rapid Run chemistry. Raw read count was 33,620,764, of which 5,054,628 remained after quality control using BBTools 38.57 (6) with bbduk options “ktrim = r k = 23 mink = 11 hdist = 1 tpe tbo qtrim = r trimq = 10 minlength = 36.”

The ONT reads were assembled with Canu 1.6 (7) with estimated genome size set to 45 Mbp. We polished the assembly with Nanopolish 0.10.2 (5) using the original ONT reads, followed by one round of PILON 1.22 (8) with the trimmed Illumina reads. Assembly contiguity and completeness were assessed with QUAST 4.5 (9) and BUSCO 3.0.0 (10), respectively. All algorithms were run with default settings unless otherwise noted. The BUSCO lineage was “sordariomyceta_odb9.”

The resulting assembly was 42,948,211 bp in length with 24 total contigs, and GC content of 52.47%. Coverage was 65.9× and 25.8× for ONT and Illumina reads, respectively. The assembly N50 was 4.4 Mbp, L50 was 5, and the largest contig was 5,591,828 bp. Completeness was estimated at 98.6% (98.4% complete and single-copy genes, 0.2% duplicates), 0.6% fragmented, 0.8% missing with 3725 BUSCOs tested.

## Data Availability

This Whole Genome Shotgun project has been deposited at DDBJ/ENA/GenBank under the accession JAULBG000000000. The version described in this paper is version JAULBG010000000. Raw sequence data are available at NCBI SRA under project number PRJNA994555. Sequences of TEF1- and RPB2 are deposited in GenBank under accession numbers OR338330 and OR338331.

## References

[1] Garnas JR, Ayres MP, Liebhold AM, Evans C. 2011. Subcontinental impacts of an invasive tree disease on forest structure and dynamics. Journal of Ecology 99:532–541. doi:10.1111/j.1365-2745.2010.01791.x

[2] Castlebury LA, Rossman AY, Hyten AS. 2006. Phylogenetic relationships of Neonectria/Cylindrocarpon on Fagus in North America. Can J Bot 84:1417–1433. doi:10.1139/b06-105

[3] Stauder CM, Garnas JR, Morrison EW, Salgado-Salazar C, Kasson MT. 2020. Characterization of mating type genes in heterothallic Neonectria species, with emphasis on N. coccinea, N. ditissima, and N. faginata. Mycologia 112:880–894. 32969327 10.1080/00275514.2020.1797371

[4] van Diepen LTA, Frey SD, Landis EA, Morrison EW, Pringle A. 2017. Fungi exposed to chronic nitrogen enrichment are less able to decay leaf litter. Ecology 98:5–11. doi:10.1002/ecy.1635 28052385

[5] Loman NJ, Quick J, Simpson JT. 2015. A complete bacterial genome assembled de novo using only nanopore sequencing data. Nat Methods 12:733–735. doi:10.1038/nmeth.3444 26076426

[6] Bushnell B. 2022. BBmap. SourceForge. Available from: https://sourceforge.net/projects/bbmap

[7] Koren S, Walenz BP, Berlin K, Miller JR, Bergman NH, Phillippy AM. 2017. Canu: scalable and accurate long-read assembly via adaptive -mer weighting and repeat separation. Genome Res 27:722–736. doi:10.1101/gr.215087.116 28298431 PMC5411767

[8] Walker BJ, Abeel T, Shea T, Priest M, Abouelliel A, Sakthikumar S, Cuomo CA, Zeng Q, Wortman J, Young SK, Earl AM. 2014. Pilon: an integrated tool for comprehensive microbial variant detection and genome assembly improvement. PLoS One 9:e112963. doi:10.1371/journal.pone.0112963 25409509 PMC4237348

[9] Gurevich A, Saveliev V, Vyahhi N, Tesler G. 2013. QUAST: quality assessment tool for genome assemblies. Bioinformatics 29:1072–1075. doi:10.1093/bioinformatics/btt086 23422339 PMC3624806

[10] Simão FA, Waterhouse RM, Ioannidis P, Kriventseva EV, Zdobnov EM. 2015. BUSCO: assessing genome assembly and annotation completeness with single-copy orthologs. Bioinformatics 31:3210–3212. doi:10.1093/bioinformatics/btv351 26059717

